# Prostate sarcoma

**DOI:** 10.1002/iju5.12578

**Published:** 2023-03-12

**Authors:** Nicholas T Ehat, Lisa Chuong, Jenna Dickman, Tom Deng, George Chang, Jonathan Hwang

**Affiliations:** ^1^ Florida Atlantic University Charles E. Schmidt College of Medicine Boca Raton Florida USA; ^2^ Medstar Urology Georgetown University School of Medicine Washington District of Columbia USA

**Keywords:** malignancy, prostate, prostatectomy, sarcoma

## Abstract

**Introduction:**

This report is intended to provide insight into the presentation, diagnosis, and treatment of prostatic sarcomas. A literature review is included to compare variables in demographics, histology, prognosis, and treatment strategies among previously reported incidences.

**Case presentation:**

In this case, we have a 72‐year‐old man who initially presented with symptomatic nephrolithiasis that led to further workup. Magnetic resonance imaging revealed an enlarged, heterogeneous prostate with a dominant mass in the left lobe. A biopsy of the prostate revealed a high‐grade, undifferentiated sarcoma in the left lobe along with concomitant adenocarcinoma in the right lobe.

**Conclusions:**

The patient underwent a radical prostatectomy, which according to existing literature remains the most effective treatment strategy. Staging is the most important prognostic indicator, making this cancer particularly dangerous as presenting symptoms are highly variable among patients.

**Abbreviations & Acronyms**
BPH: benign prostatic hyperplasia
CT: computed tomography
DRE: digital rectal examination
LMS: leiomyosarcoma
LUTS: lower urinary tract symptoms
MRI: magnetic resonance imaging
PS: prostate sarcoma
PSA: prostate‐specific antigen
RMS: rhabdomyosarcoma

**Keynote message**

This case report is intended to bring light to a rare, dangerous form of prostate malignancy. Minimal research exists on the management of prostate sarcomas. This case report will hopefully be included in larger, cross‐sectional studies that intend to discuss standardization of care for prostate sarcomas.

## Background

Prostatic sarcomas are exceedingly uncommon, constituting only 0.7% of primary malignancies of the prostate.^1^ Presenting symptoms vary among PS patients and detection is often incidental. The patient is a 72‐year‐old man who initially presented with symptomatic nephrolithiasis and was found to have an undifferentiated prostatic sarcoma. His clinical evaluation, imaging studies, histopathological findings, and treatment course are outlined.

## Case presentation

**Fig. 1:**
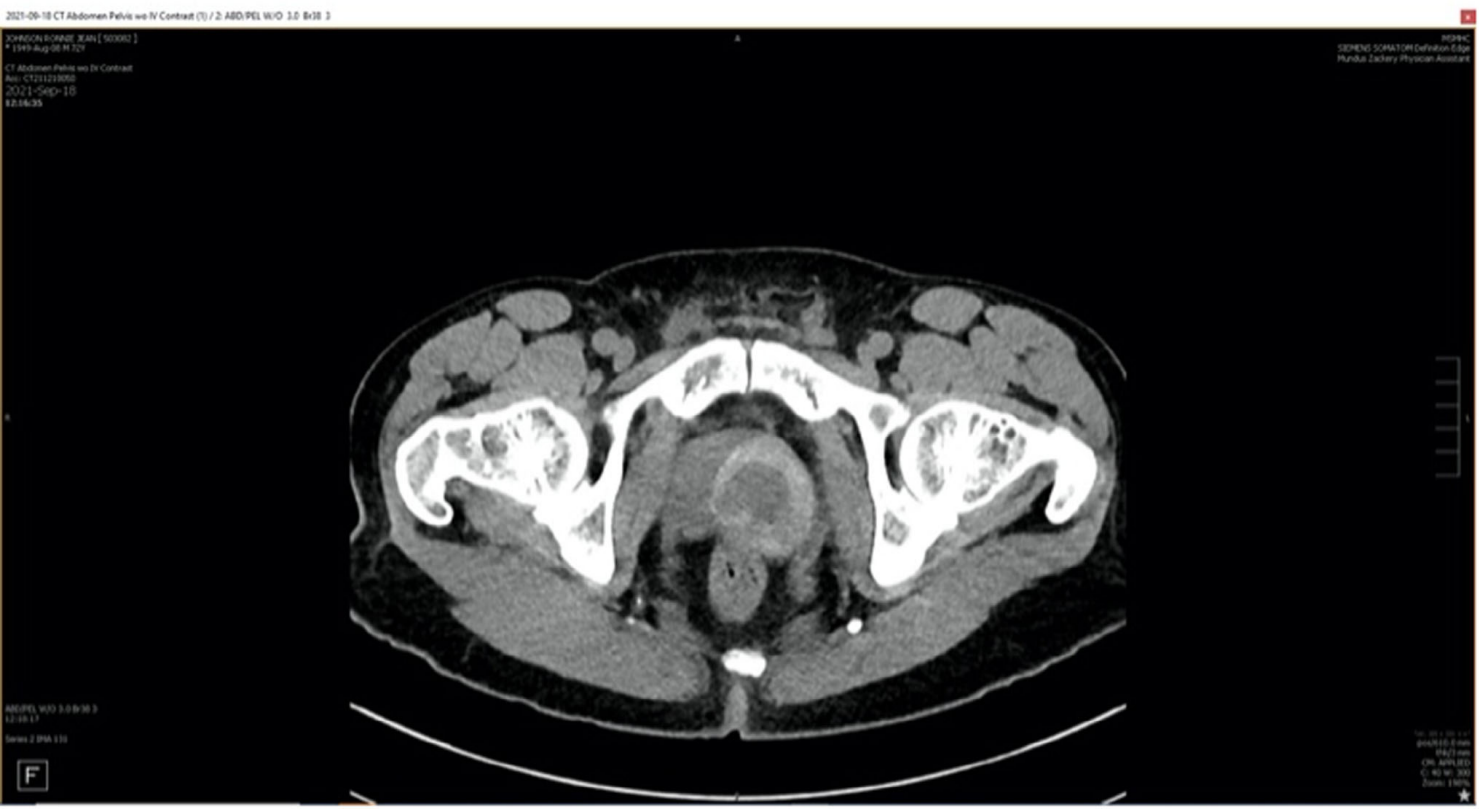
Transverse CT of the abdomen and pelvis. This image depicts a left ureteral kidney stone causing subsequent hydronephrosis of the left kidney. Additionally, a left prostatic mass can be visualized.

A 72‐year‐old male presented with left flank pain. CT of the abdomen/pelvis revealed a 3‐mm left ureteral stone with hydronephrosis and a 4‐cm left prostate mass (Fig. 1). The patient was found to have asymmetrical enlargement of the prostate gland without discrete induration or focalized mass. Basic metabolic panel was unremarkable, and a microscopic urinalysis was benign. PSA was 2.3 ng/mL on finasteride.

**Fig. 2:**
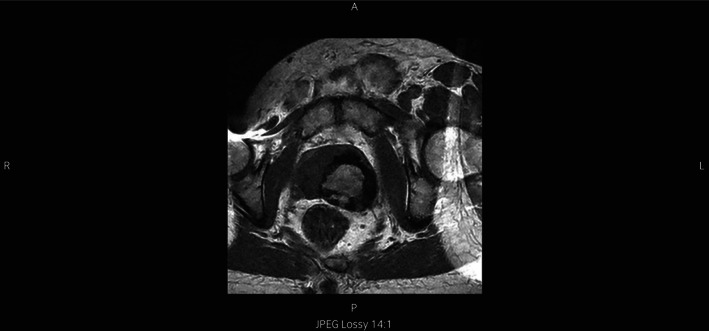
MRI of the pelvis sharply delineates a dominant mass in the left lobe of the prostate.

A subsequent MRI of the pelvis with contrast revealed an enlarged prostate with a dominant mass in the left lobe (Fig. 2). The central portion of the mass demonstrated a uniform, intermediate signal characteristic of BPH along with thick margins of decreased signal on both T1 and T2 weighted images. Prostate Imaging Reporting and Data System scoring was not assigned (not a multiparametric scan). The seminal vesicles/bladder/pelvic lymph nodes were normal. Cystoscopy showed tri‐lobar prostate enlargement without discrete lesion and normal bladder mucosa; a trans‐perineal prostate needle biopsy was positive for high‐grade undifferentiated sarcoma in the left lobe and Gleason 3 + 4 adenocarcinoma in the right lobe (Table 1). The patient's case was reviewed at a monthly multi‐disciplinary genitourinary tumor board which unanimously recommended a radical prostatectomy for a clinically organ‐confined PS with a possibility of radical cystoprostatectomy based on intraoperative findings.

**Table 1:** Clear breakdown of histopathology results from both lobes of the prostate

	Biopsy results	
	Left lobe	Right lobe
Histopathological result	High‐grade undifferentiated sarcoma	Adenocarcinoma
Gleason score	N/A	3 + 4 = 7 (Grade Group 2)
Number of biopsy cores taken	12	12
Approximate % of biopsy tissue with cancer	95%	2%
Greatest dimension of sampled cores	1.5 cm	1.7 cm
Additional information	No prostatic glands identified	Periprostatic fat and seminal vesicle invasion not identified

The patient underwent an uneventful robotic‐assisted laparoscopic non‐nerve sparing radical prostatectomy with standard template pelvic lymph node dissection through the anterior approach. Intraoperative findings were notable for a grossly organ‐confined sarcoma with unremarkable peri‐prostatic anterior fat pad, endopelvic and Denonvillier's fascia, and normal bladder neck/seminal vesicles (all included in the en bloc surgical specimen). Per tumor board recommendation, a radical cystectomy was not performed as the sarcoma appeared to be completely enclosed within the prostate gland; based on intraoperative observations, it was felt that a radical cystectomy would only lead to long‐term morbidity and poor quality of life without enhancing oncologic outcomes.

**Fig. 3:**
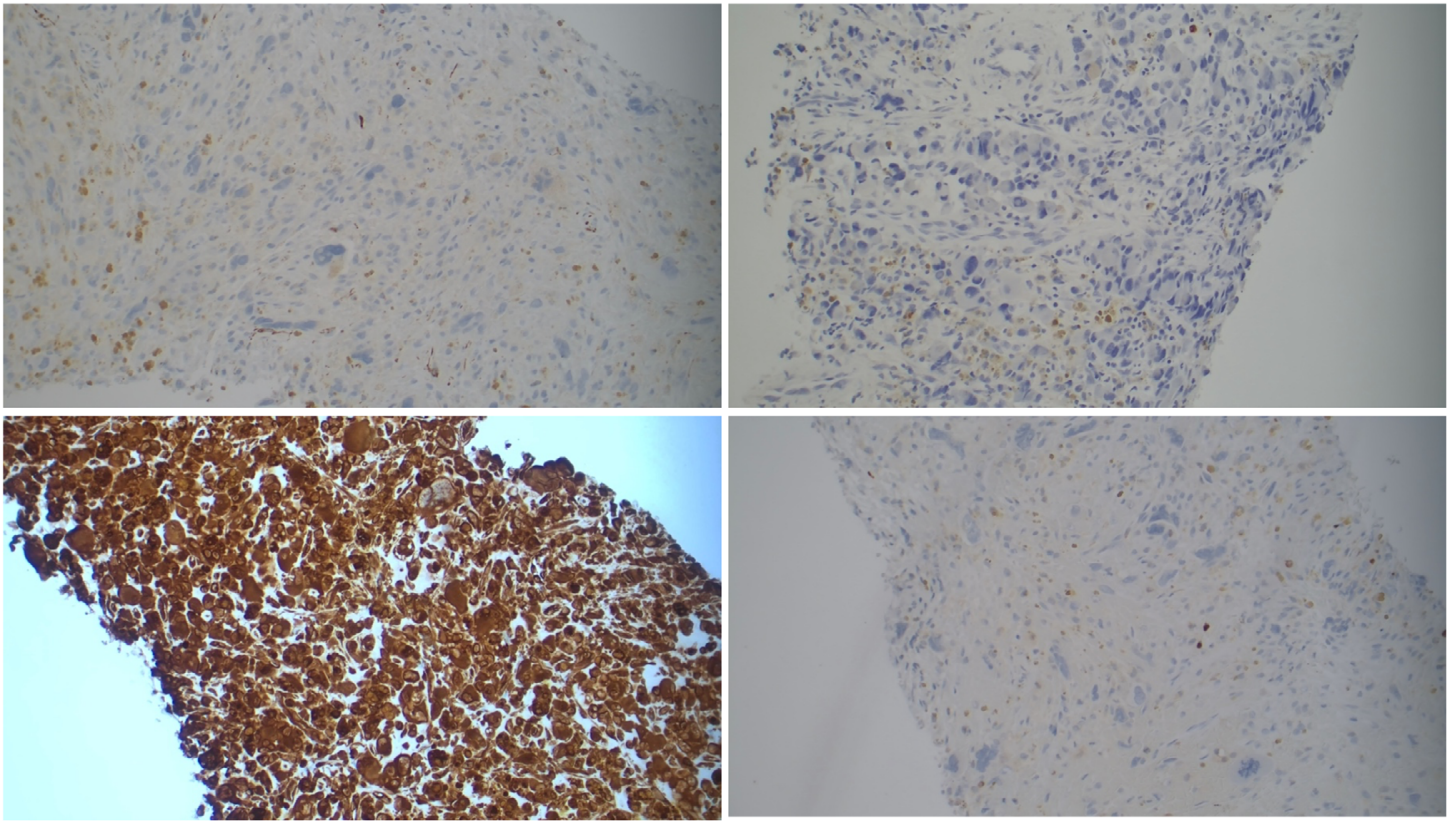
Immunohistochemistry slides. Top right, A1/A3; Bottom right, S100; top left, Desmin; bottom left, Vimentin.

The surgical pathology was notable for an organ‐confined PS (Fig. 3) and Gleason 3 + 4 (<15%) adenocarcinoma, Grade Group 2 with clear margins and uninvolved pelvic lymph nodes. The sarcoma consisted of sheets of polygonal to oval neoplastic cells with markedly pleomorphic nuclei, intranuclear pseduoinclusions, and moderate amounts of eosinophilic cytoplasm. Immunostaining showed tumor cells positive for Vimentin (*bottom left*), AE1/AE3 (*top right*), and Cam5.2 (Fig. 3). Immunostaining was negative for PSA, PSAP, GATA‐3, S100 (*bottom right*), CD34, desmin (*top left*), SMA, Myo‐D1, HMB45, and Melan‐A.

Since surgery, the patient is being followed by our medical oncology/sarcoma team with periodic CT/F‐18, FDG PET scans and at 1‐year follow‐up, the patient remains recurrence free.

## Discussion

PS are rare diagnoses that account for less than 0.7% of primary malignancies of the prostate globally.^1^, ^2^ Initial presentation most often manifests as a heterogeneously enlarged prostate on DRE or nonspecific LUTS.^1^ Screening PSA levels are frequently within normal limits. Abnormal DRE or LUTS prompt further workup with CT or MRI imaging. The final diagnosis of sarcoma, however, depends on histology and immunochemical expression.^4^

Data regarding clinical diagnosis of PS are limited to small case series and individual case reports. Through review, it appears that the median age of diagnosis is 45. However, the standard deviation is quite high due to the small number of documented cases. For example, one retrospective analysis of 38 men treated for PS had a median age of 50 (range 17–73).^1^ Another study analyzing 15 PS patients had an average age of 46.3 at diagnosis (range 22–77).^4^ Due to the small sample sizes, it is also difficult to discern additional demographics of this patient population.

Histology is highly variable among PS patients. The most common subtypes of sarcoma seen in the prostate are LMS and RMS.^1^, ^2^, ^3^, ^4^, ^5^ A unique finding in our patient was the presence of concomitant adenocarcinoma in the opposite prostatic lobe. While incidences of multiple sarcoma subtypes in the same prostate have been documented, it is rare to see sarcomatous characteristics with adenocarcinoma present concurrently. One study reviewing medical records of 20 PS patients found only 5% to have adenocarcinoma with a sarcomatous component.^6^ In our case, negative stains for desmin, SMA, and Myo‐D1 ruled out LMS and RMS. Although rare positive stains for epithelial markers (AE1/AE3 and CAM5.2) are seen, it is not uncommon for undifferentiated sarcoma.

Prognosis may be dependent upon a few factors, including tumor staging and presence of metastasis at time of diagnosis.^1^ Most importantly, negative distant metastasis has the most significant correlation with overall survival rates as well as risk of recurrence.^1^, ^2^ It seems that most PSs present early in the disease course. One study demonstrated 82.9% of the 41 patients in their review were diagnosed prior to distant metastasis.^2^ This is likely due to mass effect of the tumor causing early LUTS. Based on the rarity of occurrence of PSs, there is a lack of evidence supporting a higher cancer‐specific survival rate in a specific subtype of PS.

In general, radical resection with a negative margin contributes to the best survival rates for patients with PS.^2^ For those with metastatic disease at time of diagnosis, radical prostatectomy followed by systemic chemotherapy are the mainstay of treatment, although survival rates are often no longer than 5–7 months.^2^

To conclude, PS is an exceedingly uncommon form of malignancy. Based on its non‐specific presentation, it is often found incidentally. Staging remains the most important prognostic factor when treating a PS and surgical resection with negative margins is the mainstay of treatment.

## Author contributions

Nicholas T Ehat: Writing – original draft. Lisa Chuong: Project administration; resources; validation; writing – review and editing. Jenna Dickman: Project administration; resources; validation; writing – review and editing. Tom Deng: Data curation; formal analysis; investigation; resources. George Chang: Data curation; investigation. Jonathan Hwang: Conceptualization; data curation; project administration; resources; supervision; validation; writing – review and editing.

## Conflict of interest

The authors declare no conflict of interest.

## Approval of the research protocol by an institutional reviewer board

This manuscript has proper ethical approval and consent. IRB approval is not applicable.

## Informed consent

Written consent to publish this report was obtained from the study participant.

## Registry and the registration no. of the study/trial

Not applicable.

## Data Availability

The datasets analyzed during this study are available from the corresponding author on reasonable request.
